# Feeding Blueberry Diets to Young Rats Dose-Dependently Inhibits Bone Resorption through Suppression of RANKL in Stromal Cells

**DOI:** 10.1371/journal.pone.0070438

**Published:** 2013-08-06

**Authors:** Jian Zhang, Oxana P. Lazarenko, Jie Kang, Michael L. Blackburn, Martin J. J. Ronis, Thomas M. Badger, Jin-Ran Chen

**Affiliations:** 1 Department of Pediatrics, University of Arkansas for Medical Sciences, Little Rock, Arkansas, United States of America; 2 Arkansas Children’s Nutrition Center, Little Rock, Arkansas, United States of America; 3 Department of Pharmacology and Toxicology, University of Arkansas for Medical Sciences, Little Rock, Arkansas, United States of America; Georgia Regents University, United States of America

## Abstract

Previous studies have demonstrated that weanling rats fed AIN-93G semi-purified diets supplemented with 10% whole blueberry (BB) powder for two weeks beginning on postnatal day 21 (PND21) significantly increased bone formation at PND35. However, the minimal level of dietary BB needed to produce these effects is, as yet, unknown. The current study examined the effects of three different levels of BB diet supplementation (1, 3, and 5%) for 35 days beginning on PND25 on bone quality, and osteoclastic bone resorption in female rats. Peripheral quantitative CT scan (pQCT) of tibia, demonstrated that bone mineral density (BMD) and content (BMC) were dose-dependently increased in BB-fed rats compared to controls (P<0.05). Significantly increased bone mass after feeding 5% BB extracts was also observed in a TEN (total enteral nutrition) rat model in which daily caloric and food intake was precisely controlled. Expression of RANKL (receptor activator of nuclear factor-κB ligand) a protein essential for osteoclast formation was dose-dependently decreased in the femur of BB animals. In addition, expression of PPARγ (peroxisome proliferator-activated receptor γ) which regulates bone marrow adipogenesis was suppressed in BB diet rats compared to non-BB diet controls. Finally, a set of *in vitro* cell cultures revealed that the inhibitory effect of BB diet rat serum on RANKL expression was more profound in mesenchymal stromal cells compared to its effect on mature osteoblasts, pre-adipocytes and osteocytes. These results suggest that inhibition of bone resorption may contribute to increased bone mass during early development after BB consumption.

## Introduction

Bone development is largely influenced by nutritional status, dietary factors, body composition and the effects of weight bearing/physical activity [1]; [2]. Studies of dietary factors on bone metabolism have focused on micro-nutrients such as calcium and potassium. However, the human diet contains a complex array of factors including phytochemicals that interact with specific genes that may also be essential for bone development. It is known that fruits and vegetables are rich in phytochemicals, such as phenolic acids, flavonoids and isoflavones previously shown to be bone anabolic [3]. Moreover population-based studies have shown that fruit and vegetable intakes are independent predictors of bone mass and size in early pubertal children [4]; [5]. We have recently characterized a 10% blueberry (BB)-supplemented diet that had significant effects to promote osteoblastic bone formation in intact young male and female rats [6]. This bone anabolic effect of BB diet was suggested to be due to stimulation of osteoblastic differentiation caused by phenolic acid metabolites derived from BB polyphenols [6]. Additional inhibitory effects of BB on induction of functional tissue cell apoptosis and senescence, modulation of cell signaling, and gene expression in a variety of cell lines have been reported [7]; [8]. We hypothesize that BB diet also has an inhibitory effect on osteoclastic bone resorption and this may contribute to the significant increases in bone mass in rapidly growing rats associated with this diet.

Followed by osteoblastic bone formation, osteoclastic bone resorption is essential for maintaining an appropriate, homeostatically controlled amount of bone and shape during the entire early bone developmental period as well as in the adult skeleton [9]. Compared to osteoblasts, which are derived from mesenchymal stem cells (MSCs), osteoclasts are multinucleated cells derived from hematopoietic lineage [10]. Osteoclastogenesis, as well as activation of mature osteoclasts, is regulated by various molecular signals. RANKL (receptor activator for nuclear factor κB ligand) is an essential factor involved in both processes and has been well investigated [11]. RANKL is a secreted protein and thought to be produced mainly by osteoblasts and other cell types including mesenchymal stromal cells, osteocytes and pre-adipocytes [11]. Secreted RANKL in turn stimulates RANK (receptor activator of nuclear factor κB) signaling [12]. Osteoprotegerin (OPG) is a decoy receptor for the RANKL that binds RANKL before it has an opportunity to bind to RANK and suppresses its ability to increase bone resorption. RANKL, RANK and OPG are closely related to bone turnover and perhaps bone development. Changes in production of these RANKL/OPG mediators may underlie the development of osteopenic bone loss [13].

It is unclear whether PPARγ (peroxisome proliferator-activated receptor γ), a master transcription factor involved in adipose differentiation [14], is also affected in bone by feeding BB diet. Emerging evidence has suggested that PPARγ plays important roles in skeletal homeostasis. It has been shown that PPARγ activation suppresses osteoblast differentiation from mesenchymal stem cells by favoring adipogenesis [15]. Both *in vivo* and *in vitro* studies have shown that over-expression of PPARγ in osteoblastic cells negatively regulates bone mass and inhibits osteoblast differentiation [16]; [17]. Moreover, a recent study has revealed that PPARγ also plays an independent role to promote osteoclast differentiation from hematopoietic stem cells, and loss of PPARγ function in mouse hematopoietic lineages causes osteoclast defects [18]. PPARγ directly potentiates RANKL induction of c-Fos, an essential mediator of osteoclastogenesis [18]. The current study was designed to determine if a BB supplemented diet had a dose-dependent effect on bone accretion in young rats. In addition, inhibitory effects of BB diet on bone resorption through inhibition of RANKL and PPARγ expression in bone were investigated.

## Materials and Methods

### Animals and Diets

Female Sprague-Dawley rats were purchased from Harlan Industries (Indianapolis, IN) at age 24 days (PND24) and were randomly divided into four groups. One group of rats received control AIN-93G diet and the other three groups of rats received a different dose of blueberry (BB) supplementation (1%, 3% and 5%). Freeze-dried whole BB (Vaccinium angustifolium) powder (Hi-Actives Wild Blueberry) was kindly provided by VDF/FutureCeuticals, Momence, IL. AIN-93G supplemented with freeze-dried BB powder was made by Harlan Teklad (Madison, WI). The diets contained the National Research Council nutrient recommendations and the same calcium and phosphorus levels. The compositions of the BB diets are listed in Table 1. Rats were individually housed in an Association for Assessment and Accreditation of Laboratory Animal Care-approved animal facility at the Arkansas Children’s Hospital Research Institute with constant humidity and lights on from 06∶00–18∶00 hrs at 22°C. All animal procedures were approved by the Institutional Animal Care and Use Committee at University of Arkansas for Medical Sciences (UAMS, Little Rock, AR). The approval ID for this study is 2473. After 31 days of diet, all animals were sacrificed and tibia bones were taken and sera were collected. Food intake was measured daily from PND51 to PND55, experiment day 27 through 31 of diet. Body composition of experimental rats was assessed via NMR (EchoMRI-50–500, Echo Medical Systems, Houston, TX) as described previously [19].

**Table 1:** Nutrition Composition of Blueberry Diets.

Formula (g/Kg)	TD.10677 1% BB Diet	TD.10678 3% BB Diet	TD.10679 5% BB Diet
Casein	199.6	198.9	198.2
L-Cystine	3	3	3
Corn Starch	388.4	370.1	351.9
Maltodextrin	132	132	132
Sucrose	100	100	100
Corn Oil	69.9	69.7	69.5
Cellulose	49.586	48.786	47.886
Mineral MixAIN-93G-MX (94046)	35	35	35
Vitamin MixAIN-93-VX (94047)	10	10	10
Choline Bitartrate	2.5	2.5	2.5
TBHQ, antioxidant	0.014	0.014	0.014
**Blueberry, powder**	**10**	**30**	**50**

The bone anabolic effects of a 5% BB diet (VDF/FutureCeuticals) was also evaluated using a TEN (total enteral nutrition) rat model which has been well described previously in our lab [20]; [21]. Briefly, PND25 rats were surgically implanted with an intragastric cannula and 5% BB containing liquid experimental diets were infused 7 days later following acclimation to surgery. Liquid diets were formulated to contain the nutrients recommended for rats by the National Research Council. The control liquid diet contained 27% protein, 20% fat (corn oil), and 48% carbohydrate, the diet composition was previously published [22]. The infusion rate of TEN diets was adjusted daily in order to match the weight gain of TEN rats to that of the *ad libitum*-fed control group. The average amount of diet consumed by TEN animals was 346 Kcal per kilogram of body weight. Rats were infused TEN diets from 6 PM to 8 AM (lights off 7 PM to 7 AM) for 4 weeks. At the completion of the experiment, rats were anesthetized by injection with 100 mg Nembutal/kg body weight (Avent Laboratories, Avant, OK, USA), followed by decapitation, and serum, left and right tibia, femur, and gonadal and abdominal fat were collected.

### Bone Peripheral Quantitative Computerized Tomography (pQCT) and Histology

pQCT was performed on formalin fixed left tibia for bone mineral density measurement using a STRATEC XCT 960 M unit (XCT Research SA, Norland Medical Systems, Fort Atkins, WI) specifically configured for small bone specimens. Software version 5.4 was used with thresholds of 570 mg/cm^3^ to distinguish cortical bone and 107 mg/cm^3^ to distinguish trabecular from cortical and sub-cortical bone. Tibial bone mineral density (BMD) and bone mineral content (BMC) were automatically calculated. The position for pQCT scanning was defined at a distance from proximal tibia growth plate (1 mm below) corresponding to 7% of the total length of the tibia. Distance between each scanning was 1 mm, total of 5 scans (five slices) were carried out. Data were expressed as the mean of three contiguous slices with the greatest trabecular bone density. Vertebrae were taken from rats from each diet group. After cleaning, number five lumbar vertebra was fixed in formalin, and decalcified for histology. Standard TRAPase staining (Sigma, procedure No. 386) was performed on unstained bone sections.

### Cell Cultures

Bone marrow-derived mouse stromal cell line ST2 cells were from RIKEN CELL BANK (Ibaraki, Japan), bi-potent mouse myoblast C2C12 cells were purchased from American Type Culture Collection (ATCC; Manassas, VA, http://www.atcc.org) and murine bone marrow osteoblastic cell line OB6 cells were derived in the endocrinology laboratory at University of Arkansas for Medical Sciences [23]. Cells were cultured in α-MEM (Invitrogen, Carlsbad, CA) supplemented with 10% fetal bovine serum (FBS) (Hyclone, Logan, UT), penicillin (100 Units/ml), streptomycin (100 µg/ml), and glutamine (4 mM). As described previously [7], murine long bone-derived osteocytic cell line MLO-Y4 cells were provided by the endocrinology laboratory at University of Arkansas for Medical Sciences [24] and murine origin pre-adipocyte 3T3-L1 cells were purchased from ATCC. MLO-Y4 and 3T3-L1 cells were cultured in similar cell culture medium as above and grown in culture plates coated with collagen and gelatin, respectively. Neonatal rat calvarial cells were isolated from 3 days old pups from control diet dams using sequential collagenase digestion using a method described previously (7). Cells were seeded in 12-well cell culture or 24-well plates at appropriate density of cells per well. At 80% confluency, cells in 12-well plates were treated in the presence of 2.5% rat serum for 3 days followed by collection of RNA for real-time PCR.

### RNA Isolation, Real-Time Reverse Transcription-Polymerase Chain Reaction

RNA from bone tissue homogenized following aspiration of bone marrow cells or RNA from cultured cells were extracted using TRI Reagent (MRC Inc., Cincinnati, OH) according to the manufacturer’s recommendation followed by DNase digestion and column cleanup using QIAGEN mini columns [25]. Reverse transcription was carried out using an iScript cDNA synthesis kit from Bio-Rad (Hercules, CA). Real-time RT-PCR was carried out using SYBR Green and an ABI 7000 sequence detection system (Applied Biosystems, Foster City, CA), gene expression data were normalized to the housekeeping gene GAPDH. All primers for real-time PCR analysis used in this report were designed using Primer Express software 2.0.0 (Applied Biosystems), and are listed in Table 2.

**Table 2:** Real-Time Reverse-Transcription Polymerase Chain Reaction (RT-PCR) Primer Sequences.

Gene	Forward Primer	Reverse Primer
Rat		
RANKL	TGGGCCAAGATCTCTAACATGA	TCATGATGCCTGAAGCAAATG
OPG	GTGTGTCCCTTGCCCTGACTAC	GTTTCACGGTCTGCAGTTCCTT
PPARγ	CCAAGTGACTCTGCTCAAGTATGG	GTCATGAATCCTTGTCCCTCTGA
TRAP	CGCCAGAACCGTGCAGA	TCAGGCTGCTGGCTGAC
GAPDH	TGAGGTGACCGCATCTTCTTG	TGGTAACCAGGCGTCCGATA
BMP2	ACTTTTGGCCACGACGGTAA	TGCCTTTTGCAGCTGGACTT
ALP	TGAATCGGAACAACCTGACTGA	TTCCACTAGCAAGAAGAAGCC TTT
Osteocalcin	AAGCCCAGCGACTCTGAGTCT	GCTCCAAGTCCATTGTTGAGGTA
Osterix	AACAGCCCTGGGAAAAGGAGGA	GGAGACCATTGGTGGTGGAGA
Mouse		
PPARγ	GCTTCCACTATGGAGTTCATGCT	CCGGCAGTTAAGATCACACCTAT
RANKL	AACTGGTCGGGCAATTCTGA	GGGTTCGACACCTGAATGCT
GAPDH	GTATGACTCCACTCACGGCAAA	GGTCTCGCTCCTGGAAGATG

### Western Blotting

Right tibial bone tissue proteins were extracted for Western immunoblot analysis using cell lysis buffer as described previously [6]. Western blots were performed using standard protocols [6]. The following primary antibodies were used: beta-actin, mouse, monoclonal (Sigma); RANKL, rabbit, polyclonal; PPARγ, rabbit, polyclonal (Abcam). Secondary antibodies were purchased from Santa Cruz Biotechnology. Blots were developed using chemiluminescence (PIERCE Biotechnology) according to the manufacture’s recommendations. Quantitation of the intensity of the bands in the autoradiograms was performed using a VersaDoc™ imaging system (Bio-Rad).

### Characterization and Quantification of Polyphenol-derived Phenolic Acids in Animal Serum using High-performance Liquid Chromatography/Mass Spectrometry (LC/MS)

Sera from control or BB diet–fed rats were processed with Sep-Pak C18 SPE (Waters, Pittsburgh, PA, USA) cartridge as follows: The cartridge was washed with 3 mL of methanol, followed by equilibration with 3 mL of 0.2% formic acid aqueous solution. Serum (100 µL) was loaded onto the cartridge. The cartridge was washed with 3 mL of 0.2% formic acid aqueous solution, and total phenolic acids were recovered with 0.2% formic acid–methanol solution. The methanol solution was dried under N2 flow and re-dissolved in 200 µL of 0.2% formic acid– methanol solution for phenolic acid analysis. Characterization and quantification of phenolic acids were carried out using an Agilent 1100 HPLC system (Agilent Technologies, Santa Clara, CA, USA) coupled with a 4000 Q TRAP mass spectrometer (Applied Biosystems) according to a method described previously [26].

### Statistical Analyses

Data were presented as means ± standard error. An omnibus test comparing treatment groups was performed using Kruskal-Wallis One-Way ANOVA. Significant tests were followed by comparing each BB group to the control group and adjusting for multiple comparisons while maintaining the overall experiment-wise error rate at p = 0.05 (adjusted p-value for significance = 0.008) [27]. Dose response was assessed using Cruick’s non-parametric test for trend [28]. All statistical analyses were performed using Stata 12.0 (Stata Corporation, College Station, TX).

## Results

### BB Diet does not Affect Food Intake, Weight Gain and Overall Health of the Rats

**Figure 1:**
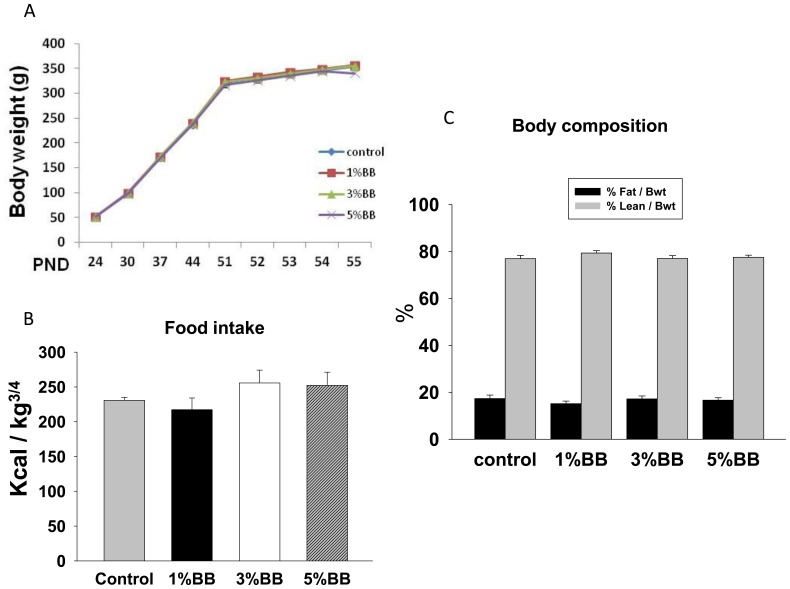
Body weight gain and composition in BB diet rats. (A) Body weight gains were monitored once a week and then daily during the last week. (B) Average food intake shows no differences among non-BB diet and 1, 3, 5% BB diet animal groups. (C) Rat NMR scan shows similar % of fat mass per body weight and % of lean mass per body weight among non-BB diet and 1, 3, 5% BB diet animal groups. Bars represent means ± SEM; n = 10 animals/group.

Postnatal day (PND) 24 male rats were fed different doses of BB diet or AIN-93G control diet for 30 days. Body weights of the rats were measured weekly and then every day the week prior to sacrifice. Weight gain in the 5% BB diet group was slightly lower but there were no significant differences among the experimental groups (Figure 1A). Food intake was recorded with no significant differences detected among the 4 groups (Figure 1B). Using NMR, body composition of rats was determined. Control diet animals and rats fed with different doses of BB supplementation had strikingly similar fat and lean content (Figure 1C). These results suggested that 1 to 5 percent BB diet does not affect food intake, weight gains, or total body fat in young rats.

### Dose-dependently Increased Bone Mass in BB Diet Rats

**Figure 2:**
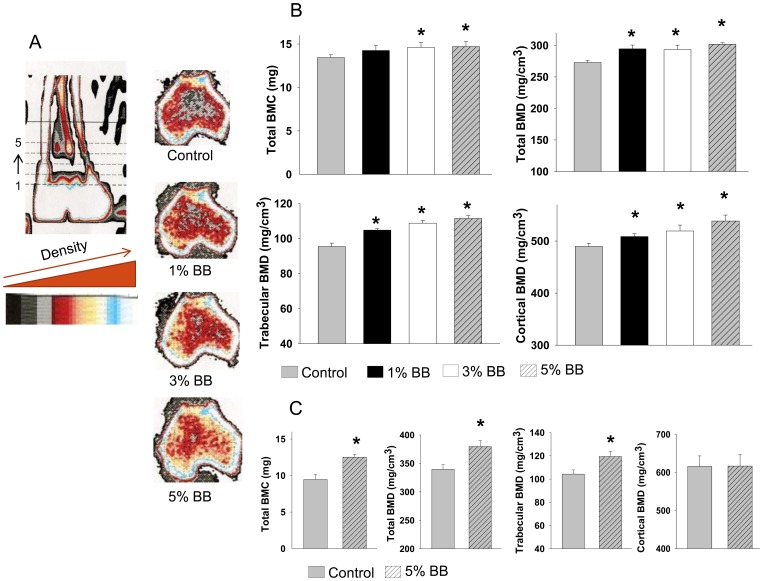
Bone mass in BB and non-BB diet rats. (A) Scout view of rat tibia using peripheral quantitative computer tomography (pQCT). Five consecutive slices (1 through 5, 1 being most distal) from each tibia were scanned using a STRATEC XCT Research SA+pQCT machine in a blinded fashion. Each slice was separated by 1 mm. software (v. 5.4) thresholds of 570 mg/cm^3^ to distinguish cortical bone and 214 mg/cm^3^ to distinguish trabecular from cortical and sub-cortical bone were used in analyzing pQCT scans slices analyzed presents those closest to the tibial growth plate. Representative tibial pQCT scans (slice 3) from control and three different BB diet rats are shown in this panel. Color changes from blue, yellow, red to gray represent decreases in bone density. (B) pQCT from four different groups of rats with BB or non-BB diet feeding. Five consecutive slices (start from about 1 mm below tibial growth plate) from each rat tibia were scanned, and data were presented after combining slice 2 and 3. Tibial trabecular bone mineral density (BMD), tibial cortical BMD, total tibial BMD and total tibial bone mineral content (BMC) were presented. (B) Tibial pQCT scan data from non BB and 5% BB diet rats with TEN (total enteral nutrition) feeding. Bars represent means ± standard error; n = 10 animals/group. *, p<0.05 versus control by *t* test. Dose response was assessed using Cruzick’s non-parametric test for trend.

To determine whether there is a dose-dependent effect of BB diet on bone quantity, standard pQCT scan beginning from 1 mm below the growth plate was carried out on tibial bones (Figure 2A). Results from pQCT analysis showed that total bone mineral content (BMC), trabecular bone mineral density (BMD) and cortical BMD were all dose-dependently increased in the BB diet groups compared to control rats (P<0.05) (Figure 2B). While the dose-dependent effects of BB diet on total BMD appeared less striking, even the 1% BB diet had a positive effect on bone mass in this rat model (P<0.05)(Figure 2B). We used a total enteral nutrition rat (TEN) model to further examine and confirm the effect of 5% BB diet on bone in a different animal model. The TEN model is a rat model we have studied extensively in our laboratory in which rat food and caloric intakes are precisely controlled [21]; [22]. Using this rat model, pQCT analysis confirmed that total BMC, total BMD and trabecular BMD were all increased in 5% BB-fed rats compared to non-BB diet control rats (P<0.05) (Figure 2C). However, no differences were found in cortical BMD between the two groups (Figure 2C).

### BB Diets Inhibit Osteoclast Differentiation Associated RANKL/OPG Pathway in Bone

**Figure 3:**
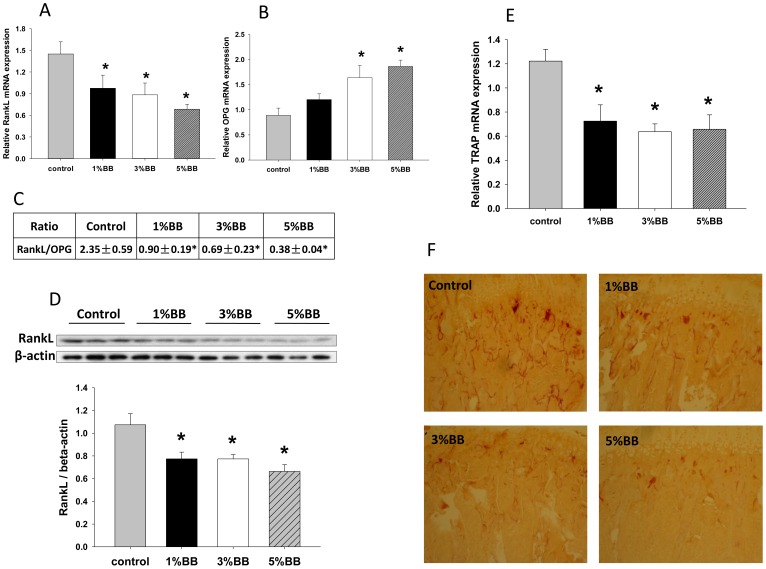
BB diets inhibit RANKL expression in bone. (A) Real-time PCR shows relative RANKL gene expression in bone from four different diet rat groups. (B) Real-time PCR shows OPG gene expression in bone from four different diet rat groups. (C) Table under the bar graph shows RANKL/OPG ratio of their gene expression in bone. (D) Western blot shows RANKL protein expression in bone from four different diet rat groups. Bar graph under the blots is quantitative data of relative RANKL intensity normalized to β-actin from western blots. (E) Real-time PCR shows relative TRAP gene expression in bone from four different diet rat groups. (F) Representative of TRAPase staining pictures from four different diet rat groups. Number 5 vertebrae bone section was stained with TRAPase, red shows the positive cells. Total RNA and protein were isolated from tibia of each animal after bone marrow aspiration for RT-PCR and western blot. For western blots, 3 to 4 animal samples were randomly pooled to one for a total of three samples per group on western blot. Data are means ± standard error; n = 10 animals/group. *, p<0.05 versus control. Dose response was assessed using Cruzick’s non-parametric test for trend.

We previously reported a robust effect of a 10% BB diet on osteoblastic bone formation in a young rat model [6]. Although osteoblastic bone formation may be dominant during early development, osteoclast involved bone resorption processes are thought to be critical in keeping bone mass and shape at appropriate levels during the entire lifespan. Our previous study suggested a possible additional effect of BB diet on osteoclasts [6]. In this regard, we therefore studied the effect of BB diet on bone resorption. It is known that RANKL is an essential factor for osteoclast differentiation and activity, and OPG binds to RANKL to sequester it. The RANKL/OPG ratio is usually looked at as a final mediator of osteoclastogenesis. The effect of BB diets on RANKL and OPG expression in bone was investigated by qRT-PCR and western blot. Both the mRNA and protein expression of RANKL were significantly suppressed after feeding BB diet for 30 days (Figure 3A and 3D). While RANKL protein expression in bone was significantly lower in all 3 levels of BB supplementation rats compared to control rats, the inhibitory effect of BB diet on RANKL mRNA expression tended to be dose-dependent (Figure 3A). This effect of BB diet on RANKL mRNA expression was inversely correlated with the expression of OPG (Figure 3B). Interestingly, the ratio of these two genes was dose-dependently decreased in BB diet fed rats (Figure 3C). TRAP (tartrate-resistant acid phosphatase) gene expression in bone was also measured using qRT-PCR. TRAP is expressed mainly by osteoclasts and therefore, used as a marker for osteoclast activity. As we expected, BB diets inhibited TRAP gene expression in bone, 1, 3 and 5 percent BB supplemented diets significantly suppressed gene expression (Figure 3E). In accordance with these data, TRAPase staining using *in vivo* bone sections prepared from vertebrae showed decreased TRAPase positive cells in bone from 1, 3 and 5 percent BB diet groups compared to control diet animals (Figure 3F).

### BB Diets Inhibit PPARγ Expression in Bone

**Figure 4:**
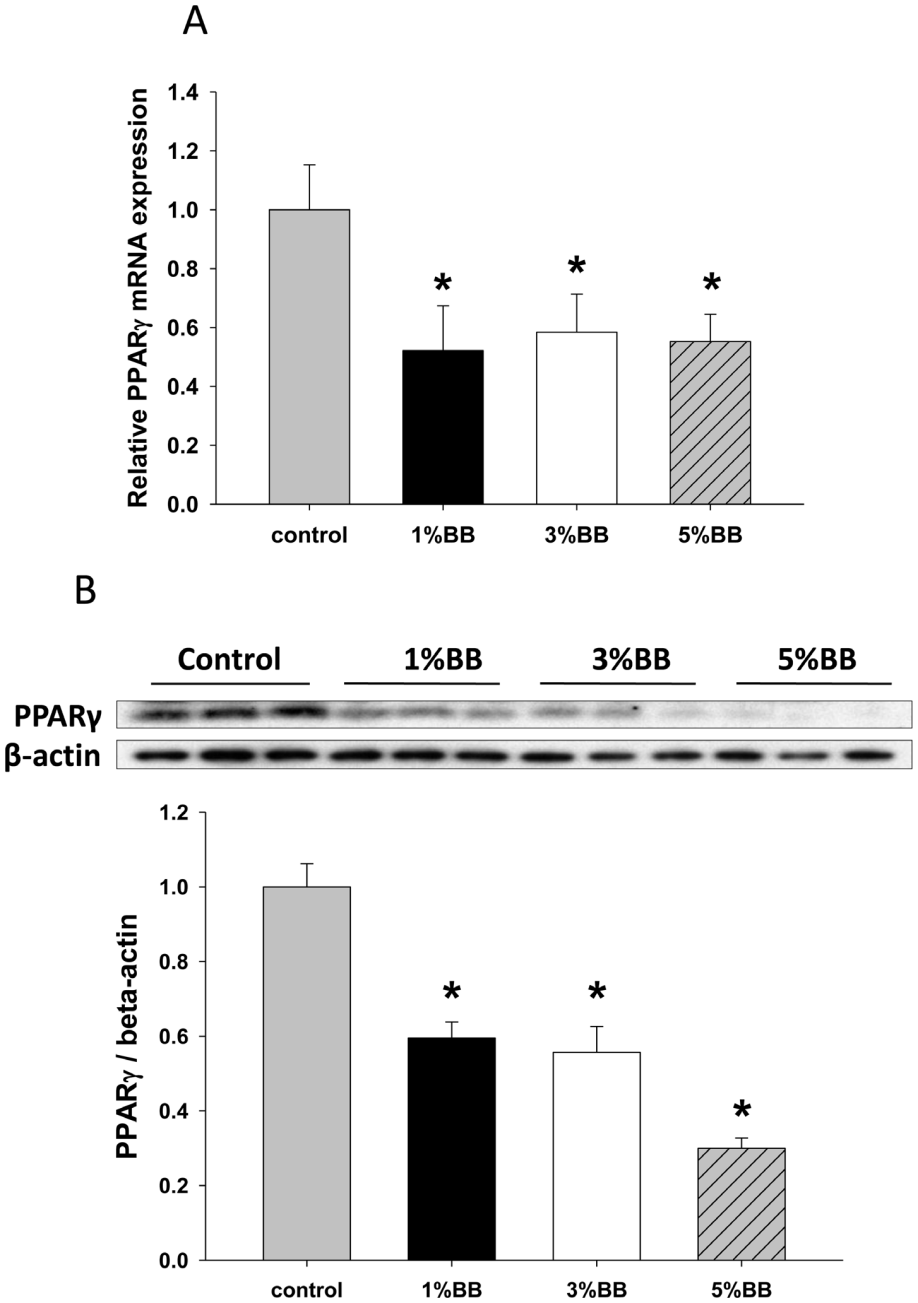
BB diet down-regulates PPARγ expression in bone. (A) Real-time PCR shows PPARγ gene expression in bone from four different diet rat groups. (B) Western blot shows PPARγ protein expression in bone from four different diet rat groups. Bar graph under the blots is quantitative data of relative PPARγ intensity to β-actin of bands from western blots. For western blots, 3 to 4 animal samples were randomly pooled to one for a total of three samples per group on western blot. Data are means ± SEM. n = 10 animals/group. *, p<0.05 versus control.

**Figure 5:**
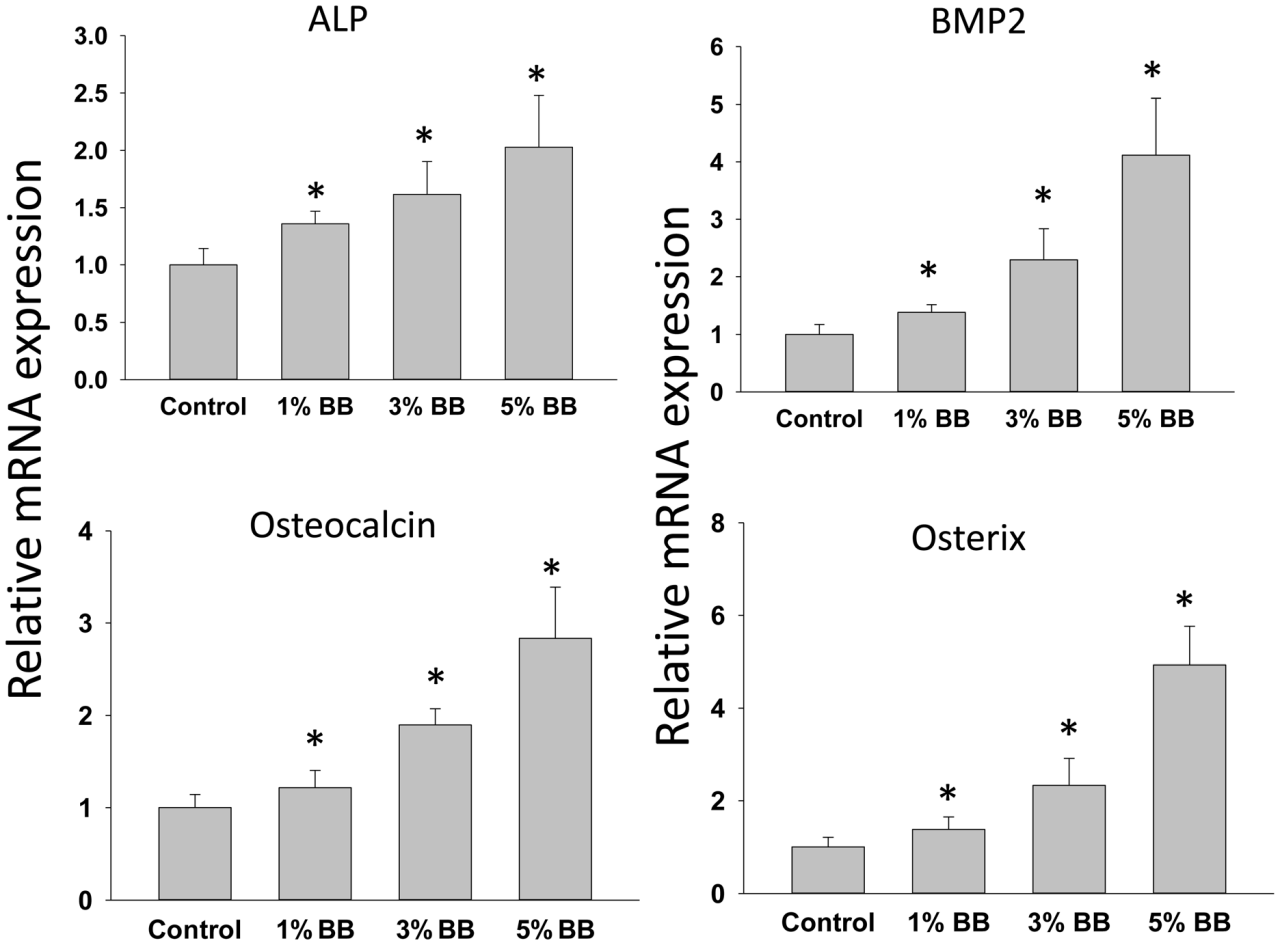
Gene expression of bone formation markers. Real-time PCR shows bone formation gene expression in bone from four different diet rat groups. ALP, alkaline phosphatase; BMP2, bone morphogenetic protein 2. Data are means ± SEM. n = 10 animals/group. *, p<0.05 versus control.

It has been shown that PPARγ has the ability to promote osteoclast differentiation from hematopoietic stem cells by directly potentiating RANKL [18]. To further elucidate whether PPARγ is involved in the inhibitory effect of BB diet on bone resorption, we measured the expression of both mRNA and protein levels of PPARγ in bone. We found that the mRNA and protein levels of PPARγ were decreased in bone in all BB diet rat groups compared to those in bone from control rats (Figure 4A and 4B). Similar to RANKL expression, PPARγ mRNA expression was significantly lower in all 3 different percentages of BB supplementation rats compared to control rats, the inhibitory effect of BB diet on PPARγ protein levels also tended to be dose-dependent (Figure 4B). The significant effect of BB diets on transcript levels of bone resorption markers led us to check if bone formation markers were affected. The expression of these bone formation markers including ALP, BMP-2, osteocalcin and osterix were all significantly increased in BB diet groups compared to those from control diet group (Figure 5).

### Bone Marrow Stromal Cells and Pre-adipocytes may be Primary Targets for the Effect of BB Diets

**Figure 6:**
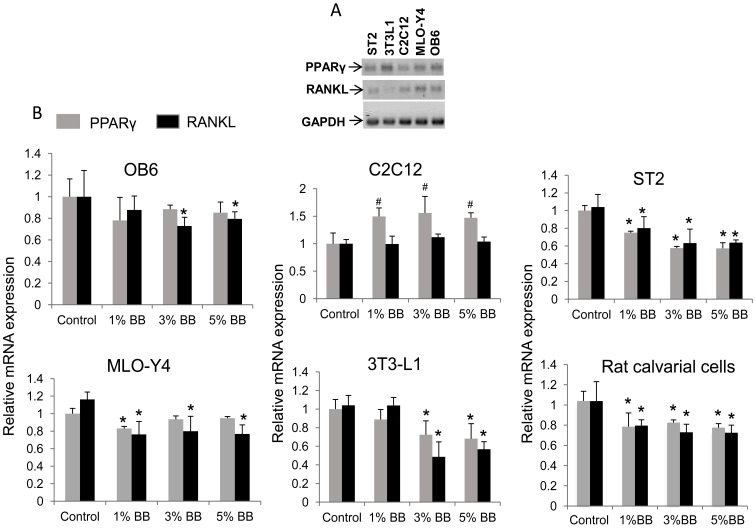
RANKL and PPARγ gene expression in different cell lines after treatment of cells with rat serum.

**Figure 7:**
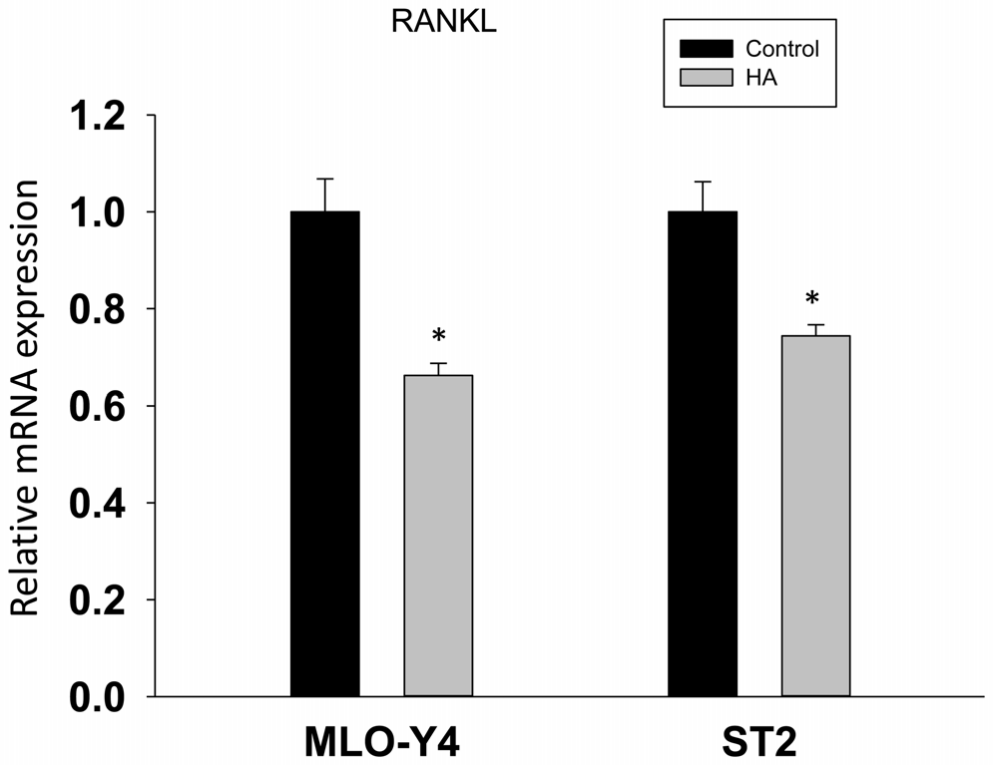
Effect of hippuric acid (HA) on RANKL gene expression in osteoblastic and osteocytic cells. MLO-Y4 and ST2 cells were treated with HA (1 µg/ml) for forty eight hours, RNA was isolated from cells for RANKL real-time PCR analysis. Data are means ± SEM from triplicate treatment. *, p<0.05 versus control by *t* test.

RANKL is a known secreted protein and thought to be produced mainly by osteoblasts and other cell types including mesenchymal stromal cells, osteocytes and pre-adipocytes. To determine which cell type is more affected by BB diet on RANKL expression, we utilized mature osteoblast OB6 cells, bi-potential pre-osteoblast C2C12 cells, bone marrow derived mesenchymal stromal ST2 cells, osteocytic MLO-Y4 cells and committed pre-adipocyte 3T3L1 cells. We first checked endogenous mRNA expression of RANKL and PPARγ in these cell types. The expression of RANKL was relatively higher in osteocytic MLO-Y4 cells than in any other cell types. On the other hand, committed pre-adipocyte 3T3L1 cells had the highest expression of PPARγ (Figure 6A). These cells were treated with 2.5% rat serum from either BB diet rats or control rats similar to an approach we have previously published [6]; [7]. RNA was isolated and RANKL and PPARγ gene expression were analyzed using qRT-PCR. Interestingly, both RANKL and PPARγ expression tended to be dose-dependently down-regulated only in ST2 and 3T3L1 cells by treatment with 1, 3 and 5% BB diet rat serum (Figure 6B). In primary neonatal rat calvarial cells, RANKL and PPARγ expression were suppressed by treatment with 1, 3 and 5% BB diet rat serum (Figure 6B). Surprisingly, PPARγ expression was increased in C2C12 cells after BB diet animal serum treatment (Figure 6B). To determine potential bioactive compounds in the serum responsible for the effects of BB diet on bone, we characterized and identified polyphenol-derived phenolic acids in serum from rats fed either BB or control diets using HPLC/MS. We found eleven phenolic acids that were present in both the BB diet groups and control diet group. Hippuric acid (HA) and salicylic acid (SLA) were dose-dependently increased in the BB diet groups (Table 3). Considering that the concentration of HA is ug/ml level, which is a hundred times higher than SLA, we speculated that HA may have an effect on bone resorption. Indeed, when we treated both osteocytic MLO-Y4 and ST2 cells with 1 µg/ml of HA, the RANKL gene expression was significantly down-regulated (Figure 7).

**Table 3:** The concentration of eleven phenolic acids present in serum of both the control and BB diet fed rats.

	Diets			
Phenolic acids (ng/ml)	Control	1% BB	3%BB	5%BB
Vanillic acid (VA)	7.81±0.43	7.61±0.03	6.84±0.05	6.88±0.68
P-coumaric acid (4-HCA)	10.47±0.88	13.18±0.26	18.24±1.69*	20.83±5.29*
Ferulic acid (FA)	433.5±74.25	504.09±2.03	478.22±4.21	359.47±28.17
Phenylacetic acid (PAA)	44.74±6.47	38.13±1.74	25.79±1.33*	31.89±2.16
Hippuric acid (HA)	516.64±64.86	1006.41±28.43*	1558.18±16.35*	2838.49±74.31*
3-hydroxycinnamic acid (3HCA)	21.86±2.93	23.71±1.85	23.22±1.61	27.17±0.21*
2-hydroxyhippuric acid (2HHA)	0.68±0.1	0.55±0.05	0.53±0.01	0.54±0.05
Syringic acid (SGA)	3.63±0.1	1.48±0.27*	2.39±0.06*	3.52±0.28
Salicylic acid (SLA)	17.11±0.28	35.27±1.97*	43.81±2.42*	51.5±0.75*
Benzoic acid (BA)	135.44±24.54	553.05±33.96*	400.25±10.83*	639.97±9.91*
3-propionic acid (TMPA)	233.8±39.82	326.18±1.35*	227.84±22.41	379.57±5.68*

ALL phenolic acids were measured by an Agilent 1100 HPLC system,

* *P<0.05, versus control diet as determined by t-test.*

## Discussion

We have presented novel data suggesting consumption of a BB-containing diet was able to suppress bone resorption through down-regulation of RANKL in stromal cells and/or PPARγ in pre-adipocytes. These effects may significantly contribute to the acute increase in bone mass in rats associated with short term feeding of BB. Whether such an effect of BB will remain persistent throughout life and contribute to optimal peak bone mass in adulthood needs to be further investigated. It has been suggested that a 10% BB in the diet in early life has a positive effect on protection against bone loss after ovariectomy in adult female rats [29]. Though bone remodeling is a process thought to occur throughout life, it is well known that during early bone development, osteoblastic bone formation exceeds osteoclastic bone resorption resulting in actual bone accrual. During this period, the main task of osteoclasts as a bone specific macrophage is to monitor appropriate bone formation, but the optimal activity and relative number of osteoclasts required for this process are still unknown. We have shown the effect of our BB diet is mediated via suppressed expression of RANKL which is a master regulator and marker gene of osteoclast differentiation and activity. Down-regulation of the RANKL/OPG ratio and TRAP further support the observation that BB suppresses bone resorption in our rat model. RANKL is expressed in a variety of tissues and cell types [11]. Our *in vitro* studies additionally answered the question of whether the effect of BB is cell type specific. The BB diet inhibited RANKL expression in bone marrow derived mesenchymal stromal ST2 cells, but surprisingly, RANKL was less affected in osteocytes. These data suggested that in young rapidly growing rats, bone marrow stromal cells are targets for BB to regulate RANKL expression and osteoclast differentiation.

PPARγ is reported to act as a key transcription factor in energy homeostasis, cardiovascular disease, and diabetes [30]–[32]. Emerging evidence suggests that PPARγ also plays important roles in skeletal homeostasis. Since osteoblasts and adipocytes share a common original stem cell, activation of PPARγ forces mesenchymal stem cells to differentiate into pre-adipocytes rather than osteoblasts [21]; [33]. Recent studies have revealed an independent role of PPARγ in regulation of osteoclast differentiation. Wei *et.al* reported that osteoclast progenitors reside in PPARγ-expressing bone marrow cell populations [34]. Deletion of PPARγ in osteoclasts impaired osteoclast differentiation and compromised RANKL signaling [15]. Consistently, increased stimulation of PPARγ resulted in an increase of RANKL mRNA expression in rosiglitazone-treated animals [35]. In our study, the expression and activity of PPARγ in bone were significantly decreased by different doses of BB supplementation in the diet (**Figure 4**). We speculate that changing of PPARγ expression in bone and bone marrow may be the mechanism by which BB diet suppresses RANKL and osteoclast differentiation. More importantly, compared to control diet, BB diet did not affect body weight and fat content. This suggests that the BB diet probably affects PPARγ in a tissue-specific fashion. We believe that using RANKL or PPARγ gene modified animal models may answer this question.

Polyphenols are the most abundant bioactive compounds of BB and mainly include proanthocyanidins, anthocyanidins, flavones, phenolic acid and stibenes. Phenolic acids are efficiently absorbed and are one of the final metabolites derived from the other polyphenols *in vivo*. In order to identify which BB derived compounds circulating in animal serum following consumption of a BB diet has an effect on bone resorption, we characterized and identified polyphenol-derived phenolic acids in serum from rats fed either BB or control diets using LC/MS. Among the phenolic acids we analyzed, ferulic acid (FA), hippuric acid (HA), benzoic acid (BA) and 3-propionic acid (TMPA) had the highest concentrations in serum, while only HA and salicylic acid (SLA) tended to be dose-dependently increased as % BB increased in the diet. Although data presented in the current study showed that HA significantly inhibited RANKL gene expression in both osteocytic cells and ST2 cells, we will determine whether among the phenolic acids, HA is one of the more important compounds responsible for the inhibitory effect of BB diet on bone resorption *in vivo* in our future studies.

We have demonstrated that feeding a low BB diet dose-dependently increased bone mineral density and content in rats without affecting normal growth. This increased bone mineral density is associated with inhibition of bone resorption accompanied by a decreased RANKL/OPG ratio and TRAP expression. Furthermore, decreased expression and activity of PPARγ in bone may also explain the dose-dependent effects of BB diet on inhibition of bone resorption. Finally, we suggest that HA is one of the more abundant metabolites derived from BB pigments, exerting its effects, at least in part, via suppression of bone resorption.

In the current study, we demonstrated for the first time that consumption of a BB supplemented diet for only two weeks had a dose-dependent anti-osteoclastogenic activity without affecting weight gain, food intake and total body fat in young rats. This inhibitory effect of BB on bone resorption was associated with an increased bone mass compared to non-BB diet-fed rats. In our previous studies we demonstrated that 10% BB diet had the ability to promote osteoblastic bone formation in rapidly growing rodents increasing the bone mass by nearly 35% [6]. Taken together with the present study, our data suggest that the effect of BB supplemented diet on bone mass is dose-dependent. Even a diet supplemented with as low as 1% BB significantly increased trabecular bone mineral density over that of control rats. We believe that our current animal studies provide a rationale for further studies in humans exploring the effect of BB-containing diets on bone development in both children and adults. According to animal and human diet dosage conversion formula [36] and USDA (United States Department of Agriculture) National Nutrient data base, a 10% BB supplemented diet is equivalent to 171 to 200 kcal for a 65 to 70 kg human per day. While this level of BB supplementation may theoretically be consumable, a lower% BB diet may be more practical for future clinical studies and still have significant bone anabolic effects.
